# Case of an Uncommon Disease Caused by an Insect in the Liver

**Published:** 1806-01

**Authors:** 


### Case of an uncommon Disease caused by an Insect in the Liver.

DOCTOR DESFONTAINES, of Paris, attended a gentleman, aged thirty-three, who had always enjoyed good health, being of a strong and robust constitution. When the Doctor first saw him, he complained of violent pains in his stomach, and a difficult digestion, but these inconveniences were sometimes relieved by a slight diarrhœa. His appearance was pale and rather ash-coloured. In the urine, a yellow or whitish sediment was observed. His tongue was always foul, and his pulse thin and hard. From

From these appearances the doctor was led to conclude, that the complaint arose from obstructions in the intestines, and accordingly ordered diluent medicines, whey, infusions of resolvent herbs, broth, &c. After some days, an opening medicine was given, which seemed to relieve the patient a good deal. Three weeks after he was violently seized with a vertigo and head-ache, which continued without the least interruption. The pain in his stomach grew worse, and extended towards the great lobes of the liver. At the same time the patient lost his appetite, and frequently fainted; his skin was tinted of a saffron colour; his breath became very offensive, the urine thick, and of a brownish colour. Soon after he had some bilious dejections. Having taken some opening medicines, injections, bitter and stimulating medicines, Dr. D. ordered emollient cataplasms and laudanum, in order to procure him some relief. During the space of five weeks all the symptoms increased; every kind of food brought on sickness, and was thrown up again by vomiting. The fever rapidly increased, the extremities became swelled. The Doctor ordered half-a-drachm of jalap with a little calomel, without effect; the next day the same was repeated, which brought on a discharge of blood, and soon after the patient expired.

Dissection being made, all the contents of the chest were found natural; the stomach was much contracted, and its upper aperture appeared in plaits. The inner coat was red, inflamed, and in some parts gangrenous. The intestines much extended with air. The pancreas preternaturally enlarged. The vesica fellis was empty, and contained only two small bilious stones. The liver was much smaller than natural, in some parts hardened and scirrhous; in others more pale and livid. Towards the middle of the concave surface of the great lobe, a small aperture was observed of six or seven lines in diameter, entering about four or five lines deeper. This cavity was filled with a black, thick fluid, and *a living insect* crept out, of a peculiar kind, quite dissimilar to insects sometimes observed in the liver, and known under the name of hydatids of the liver. It was like a smooth caterpillar, or silk-worm, of the same thickness and shape, and four inches long. The colour was of a brownish red, the body ringled, each of these rings having a white spot, in the middle of which a hair was standing upright, as in some species of caterpillars; the lower part ended in a tail something like that of a crayfish.

This

This very singular case, reminds us of an account given by M. *Lannec* (Journal de Medicine, An. XIII. pluviose, p. 55.) where *ascarides* were found in the bilious duct of a child. The patient died suddenly, at the age of two years and a half. He was not emaciated, and all the intestines had their natural appearance, the stomach and liver excepted. The stomach was equal in size to a duck's egg, and entirely filled with white and cylindrical worms, from six lines to five inches long, which, on examination, were found to be ascarides. The internal coat of the stomach was in different places more red, but no inflammation or thickening of the internal membrane was to be observed. The ductus choledochus was more enlarged, to about half an inch diameter; the ductus hepaticus was likewise much larger; all these were filled with ascarides, and contained so little of the bilious matter that these worms were not even stained by it. The pituitous membrane of these ducts was somewhat thickened with red spots. In some parts the membrane was corroded. The ascarides were in contact with the liver, and a number of cavities were observed to be formed by them, some of which were of the size of an almond. The vesicula fellis likewise was filled with ascarides. It is very remarkable that no worms were found in the other intestines.

